# Supplementary material for: Machine Learning Prediction of Cardiac Resynchronisation Therapy Response From Combination of Clinical and Model-Driven Data
Source: Front Physiol. 2021 Dec 14;12:753282. doi: 10.3389/fphys.2021.753282 (PMC8712879; doi:10.3389/fphys.2021.753282)
Supplement: Supplementary file 1 [file Data_Sheet_1.pdf]

Supplementary Material

SUPPLEMENTARY DATA

S.1 Myocardial damage simulation in the personalized ventricular models

To create an area of myocardial damage (post-infarction scar or on-ischemic fibrosis) in personalized ventricular models we used MRI examination protocols from the patient medical history recorded by radiologists. For every patient an LV myocardial damage map was schematically drawn by an expert using a 17-segment AHA LV model, in which each segment was additionally divided into three transmural layers. In the scheme segments/layers with fibrosis or scar were shown in color. Figure S1 shows example of such schematic myocardial damage annotation for one patient from our cohort.

Each personalized LV geometry computational model was also segmented into 17x3 regions (17 segments and 3 layers) according to the AHA scheme. Corresponding regions in the model indicated on the patient's LV myocardial damage map were designated as fibrosis/infarct area (see Fig. S1 for examples). The scar regions were then simulated as an inexcitable area, and fibrosis regions were associated with a low myocardial conductivity parameter.

Figure S3 demonstrates a diagram with scar/fibrosis distribution between the AHA ventricular segments and the relative volume of the infarct/fibrosis zone in every segment of the AHA LV model. The Infarct/fibrosis volume was calculated according to the computational model. The relative volume against the myocardial volume was also defined (see Table S1 for statistics).

Figure S4 shows distribution of LV pacing electrode locations between the AHA segments derived from the CT scans and used in the ventricular models under BiV pacing. The right ventricular electrode in all patients was located in the RV apex. The distances from the LV pacing sites to the area of late activation time (LAT) in LBBB simulations and to the area of infarct/fibrosis was computed by the model segmentation (see Table S1 for the statistics).

S.2 Analysis of clinical data before and after CRT device implantation and model simulations in LBBB and BiV pacing in the patient cohort

A summary of clinical data statistics before and after CRT device implantation, CT/MRI derived data and model-driven biomarkers in LBBB and BiV pacing in the entire patient cohort is presented in Table S1. It is clearly demonstrated that all clinical indicators of the CRT outcome show an average positive response in the entire cohort. On average, QRSd is decreased by 23±14%, EDV and ESV are decreased by -18±31% and 24±36% respectively; EF is increased by 9±8%; NYHA functional class is decreased at least 1 point in 54% of patients. Mechanical dyssynchrony indices characterising asynchrony in inter- and intra-ventricular contraction show prominent average decrease in the patient cohort.

In consistency with the clinical data, the model simulations also demonstrate an average positive outcome of BiV pacing as compared to the LBBB activation (Fig. 4). Average TAT and QRSd are decreased by 45±22% and 26±17% respectively, and the latter is in good agreement with the clinical effect of BiV pacing on the QRSd. The electrical dyssynchrony indices also reveal a prominent decrease in the population of models, with the highest reduction in the inter-ventricular dyssynchrony index AT_{RVLV} by 76±24%.

However, we can see a high variability in the biomarker responses to the BIV pacing in both the clinical and simulated data. Coefficient of variation (SD/mean) in the relative change of some features is higher

than 100% (e.g. see ΔEDV, ΔESV, and Δ for mechanical inter-ventricular dyssynchrony indices in Table S1), suggesting a significantly nonuniform output among the patients.

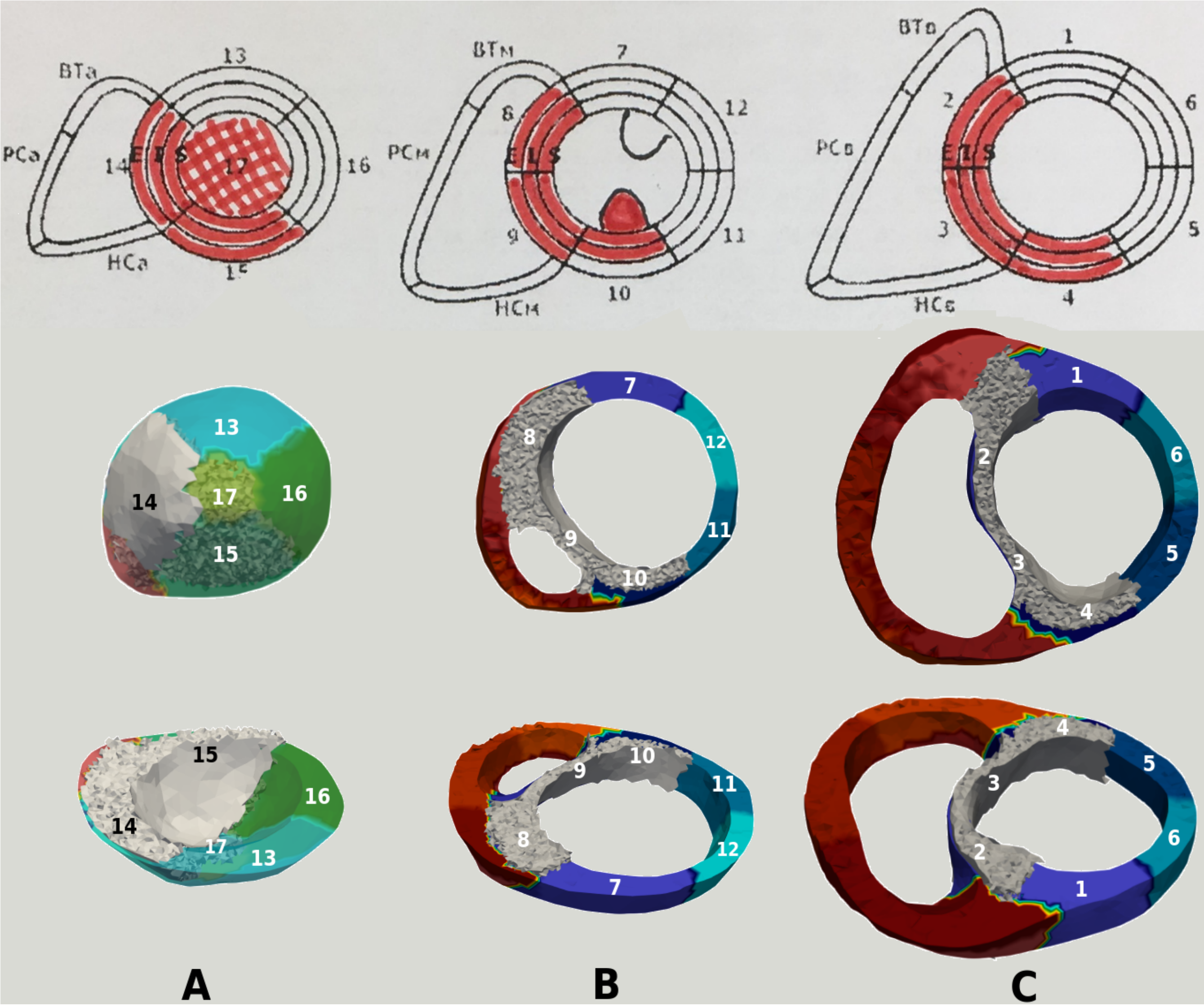

Figure S1. Top: An example of LV damage map showing apical (A), middle (B), and basal (C) regions from a 17-segmental AHA LV model labelled by a radiologist examined an MRI scan. Infarct area is colored in red. Centre and bottom: regions of a personalized ventricular model with the scar area assigned to the same LV segments as labelled in the expert map. The scar is colored in light grey. AHA LV segments with normal myocardial tissue are colored in different colors.

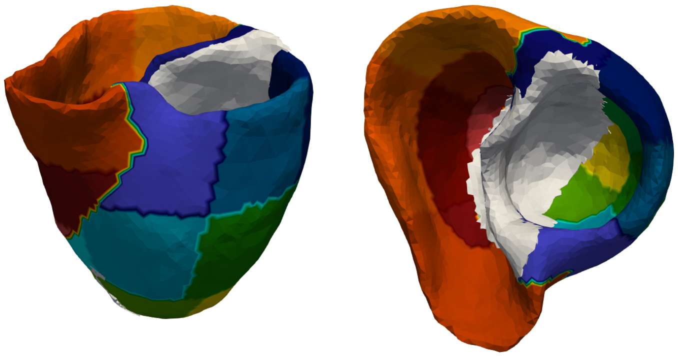

Figure S2. Personalized ventricular model of geometry segmented from CT scans. The model is segmented into colored AHA segments of normal myocardial tissue and white infarct area built upon the expert's damage map shown in Fig. S1.

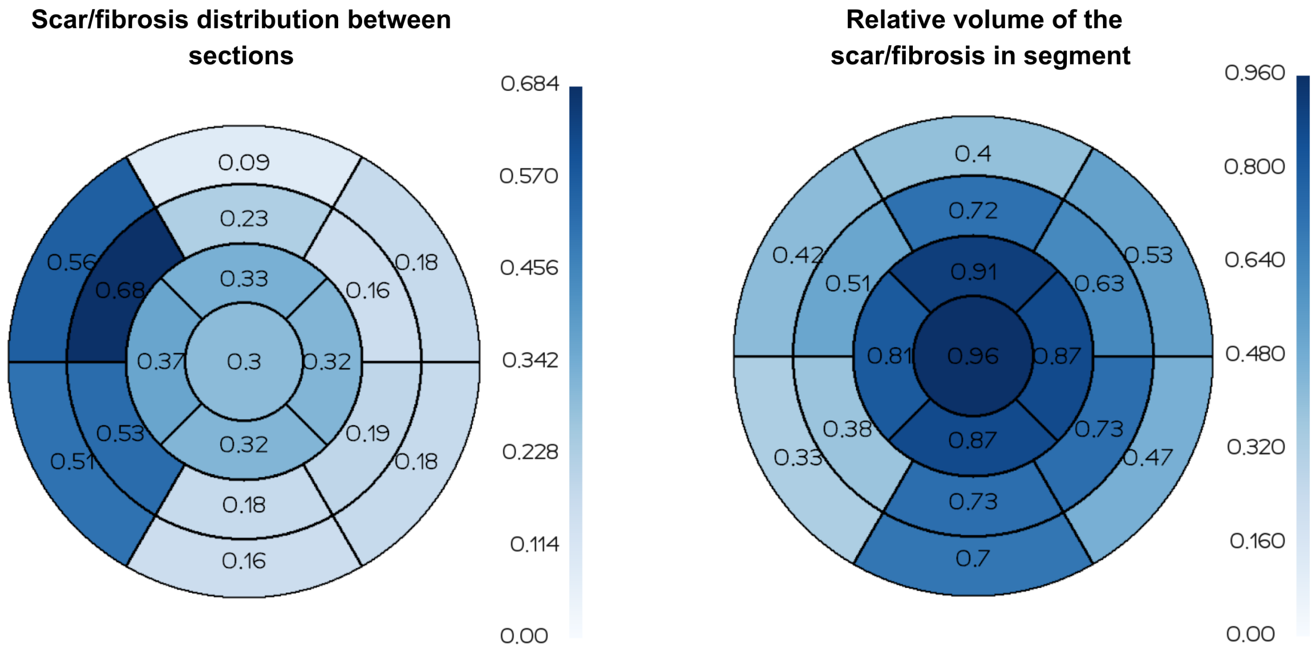

Figure S3. Left: scar/fibrosis distribution between the AHA ventricular segments. Darker colors indicate segments in which more patients have infarction/fibrosis. Numbers indicate the proportion of patients who have infarction/fibrosis in a given segment. Right: the relative volume of the infarct/fibrosis in every segment of AHA LV model. Darker colors indicate segments in which infarct/fibrosis occupies a larger volume of myocardial tissue. Numbers indicate the average proportion of injured volume from segment volume in patients with infarct/fibrosis

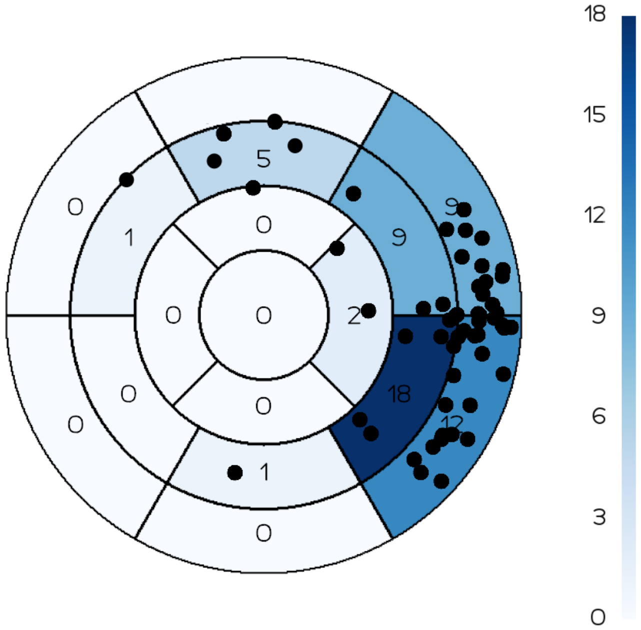

Figure S4. Distribution of LV pacing electrodes. Darker colors indicate segments with a larger number of implanted electrodes. The numbers indicate the amount of electrodes implanted in a given segment in the cohort of 57 patients

S.3 Correlations within the input (pre-operative) data allowing input dataset dimension reduction

In our hybrid input dataset, each patient was assigned an input vector of measured or simulated biomarkers potentially available before operation. The hybrid dataset contained features from different semantic blocks: clinical data of different modalities (anthropomorphic, clinical assessment/diagnosis, instrumental data from ECG, echocardiography), CT/MRI derived indices, and simulations on a personalized ventricular model in both LBBB activation and BiV mode.

First of all, we excluded all mechanical dyssynchrony indices from the hybrid dataset as there were missed values in these features. Thus, we considered 31 features per patient for further analysis. To decrease the dimension of the input vector of biomarkers, we first analyzed pair-wise correlations between the features and found pairs having high correlation coefficients (r>0.75, p<0.05, see the hit-map in Figure S5 in Supplementary Materials showing color-scaled r-values between the features). While several input features demonstrated high correlations, we excluded one feature from every pair with r>0.85. This is a conventional practice in data analysis preventing information loss caused by a possible overestimation of the correlations between features due to the sample size being rather small.

We found no high correlations between the parameteres from the data-blocks of different semantics and modalities, suggesting that data from each block should be further used for CRT response classifier development. The only exception was a high correlation between clinical QRSd_{LBBB} recorded before CRT operation and model-derived QRSd_{LBBB} in the LBBB activation mode (r= 0.84). This correlation is not surprising because personalized LBBB models were tailored using ECG data recorded during electrocardiographic imaging using the Amycard EPI-system in patients in the LBBB activation mode with

Table S1. Clinical, imaging, model data and predictive model scores for the patient cohort.

Variable	All patients (n=57)			
	**Clinical data**			
Gender (male/female)	38/19			
Age, year	63±6			
BMI	28±5			
IHD/DCM	36 (63%)/21(37%)			
History of AF	12 (21%)			
	**LBBB**	**CRT**	**Δ, %**	**P***
FC CHF :	decrease in FC 31 (54%)			
I	0 (0%)	10 (17.5%)*	10	**0.002**
II	24 (42%)	31 (54.5%)	7	0.052
III	33 (58%)	6 (10%)*	-27	**0.001**
QRSd, ms	191±24	144±19*	-23±14	**0.000**
	**Echocardiography data**			
EDV, ml	294±92	236±119*	-18±31	**0.000**
ESV, ml	217±77	159±101*	-24±36	**0.000**
EDD, mm	73±8	66±10*	-10±10	**0.000**
ESD, mm	63±9	53±12*	-15±17	**0.000**
EF, %	26±6	35±8*	9±8	**0.000**
IVD, ms (n=34)	69±19	38±18*	-42±28	**0.000**
ΔTs, ms (n=34)	85±40	65±35*	-16±65	**0.004**
SD12, ms (n=34)	32±15	24±12*	-17 ±61	**0.003**
	**CT/MRI data**			
MTV,ml	359±143			
InfarctV, ml	50±39			
InfacrtV/MTV	0.15±0.11			
DLvRv,mm	106±24			
DLvLATZ,mm	52±24			
DLvInfarct,mm	36±28			
	**Model data**			
	**LBBB**	**BiV**	**Δ,%**	**P***
TAT, ms	256±122	139±41*	-45±22	**0.000**
QRSd, ms	189±23	148±24*	-26±17	**0.000**
AT_{RVLV}, ms	98±73	22±23*	-76±24	**0.000**
$IntAV_{STLV}$, ms	104±58	34±16*	-52±46	**0.000**
mAT_{STLV}	0.36±0.09	0.28±0.13*	-8±2	**0.007**
	**Predictive model scores**			
Score by Feeny et al **?**	0.59±0.22			
MLCD score (EF10)	0.41±0.24			
MLHD score (EF10)	0.41±0.27			
MLHD score (ESV15)	0.68±0.18			

Mean±SD
P* - LBBB *vs* CRT or LBBB *vs* BiV. Comparisons between two dependent groups were made using Wilcoxon's test for quantitative data and McNimar's test for qualitative data.
Δ - Average change in indicator ΔX = X CRT - X LBBB / XLBBB or ΔX = XBiV - X LBBB / X LBBB. Δ is calculated as the absolute difference for normalized values (EF and mAT_{STLV}) and FC
BMI - Body mass index; IHD -Ischemic heart disease; DCM - Dilated cardiomyopathy; AF - Atrial Fibrilation; FC CHF- functional class of congestive heart failure; IVD - interventricular dyssynchrony; ΔTs - maximum temporary difference in peak systolic velocities between 12 LV segments; SD12 - standard deviation of the peak systolic velocities of 12 LV segments; MTV - myocardial tissue volume; DLvRv - distance between active poles of LV and RV leads; LAT - late activation time; DLvLATZ - distance between LV lead and LAT zone; DLvInfarct - distance between LV lead and infract/fibrosis area; TAT - total ventricular activation time; QRSd - maximal duration of QRS complex on 12 leads; AT_{RVLV} - difference of total LV and RV activation time; $IntAV_{STLV}$ - integral index of LV free wall and septum myocardial activation dyssynchrony; mAT_{STLV} - difference between mean activation time of LV free wall and septum; MLCD score (EF10) – ML score on the clinical data for EF10 criterion; MLHD score (EF10) - ML score on the hybrid data for EF10 criterion; MLHD score (ESV15) - ML score on the hybrid data for ESV15 criterion;

ventricular pacing turned off(see Methods Sec. for details). However, as this correlation did not overcome the threshold for data exclusion, both features were further used for classifier development.

Among the pre-operative clinical data, high positive correlations were found between LV EDV and ESV and between the corresponding linear LV dimensions EDD and ESD derived from echocardiography records before operation (r=0.97 and 0.94, respectively). The latter suggests the possibility of using either one dimensional biomarker as representative of both LV dimensions. Therefore, ESV and ESD features in each pair were excluded from the hybrid dataset used to develop a classifier.

Among the simulated features, we found high cross-correlations between the TAT/MTV and QRSd/MTV values (normalized to the survived myocardial tissue volume (MTV)) between each other in the same mode of activation and between each of them in the LBBB and BiV pacing (see numbers in Fig. S5). However, only QRSd/MTV$_{LBBB}$ and QRSd/MTV$_{BiV}$ showed a high correlation (r=0.90), which allowed to exclude the former feature from the dataset for classifier building. We found also some high correlations between simulated electrical dyssynchrony parameters and TAT under LBBB, and between the two inter-LV dyssynchrony indices under BiV pacing, but no one pair overcame the threshold for data exclusion (see Fig. S5).

Thus, we excluded 3 input features from the dataset. Finally, the hybrid dataset containing 27 input features per each of 57 patients was used further for classifier development (see the complete feature list in Figure 4).

S.4 Correlations within the output (post-operative) data

We performed a similar pairwise cross-correlation analysis for post-operative clinical features derived from ECG and echocardiography recordings, which are conventionally used to characterise CRT response in patients (see Figure S6 in Supplementary Materials). The CRT outcome dataset contains QRSd, EDV, ESV, and EF a year after CRT device implantation and their relative change against pre-operative values. High correlations (r>0.75) were found between EDV$_{CRT}$ and ESV$_{CRT}$, and between ΔEDV$_{CRT}$ and ΔESV$_{CRT}$. These findings support stronlgy the criteria we chose to identify CRT responders in our patient cohort using a separate analysis of either ΔEF$_{CRT}$ or ΔESV$_{CRT}$, as these showed moderate dependence on each other (r=-0.61) and low-to-moderate dependence on other output features (Fig. S6).

S.5 Correlations between input and output features

Before building CRT response classifiers using ML approaches we analysed pairwise cross-correlations between input features and post-operative CRT outputs (see the correlation hit-map in Figure S7 in the Supplementary Materials). Our aim was to find potentially most predictive features from our input hybrid dataset and compare the feature importances assessed by linear regression with those selected by ML classifiers. The highest correlation (r=0.81) is seen between simulated QRSd$_{BiV}$ in BiV mode and clinical post-operational QRSd$_{CRT}$. The correspondence between the simulated and clinical features reflects a good quality of personalized models fitted to post-operational ECG data recorded during BiV pacing by means of electrocardiographic imaging (Amycard system), and justifies the use of model-driven features in BIV mode for CRT response prediction. A high correlation was also found between clinical QRSd$_{LBBB}$ before operation and ΔQRSd$_{CRT}$ (r=-0.74) suggesting higher electrical synchronization in patients with initially shorter QRSd.

Other input-output features show either low or moderate correlations (see Fig. S7). In particular, post-operational ΔEF$_{CRT}$, which we used to define CRT response, correlates with clinical BMI and pre-operational EF$_{LBBB}$, and some model-derived features: distance from the LV pacing site to the late activation zone, normalized QRSd/MTV$_{LBBB}$, TAT/MTV$_{BiV}$ and QRSd$_{BiV}$. At the same time, ΔESV$_{CRT}$ showed correlations only with EDV$_{LBBB}$ and ESV$_{LBBB}$ and with the distance from LV pacing site to the area of LAT. For some input features we found no correlations with any of the output indicators, e.g. for simulated LBBB indices of ventricular electrical dyssynchrony.

Summarising the results of our analysis, we found a variety of relationships between clinical data before and after CRT, as well as between model-driven indices and clinical indicators of response. We hypothesise that combining clinical and simulated features can significantly improve prognostic models of the CRT response.

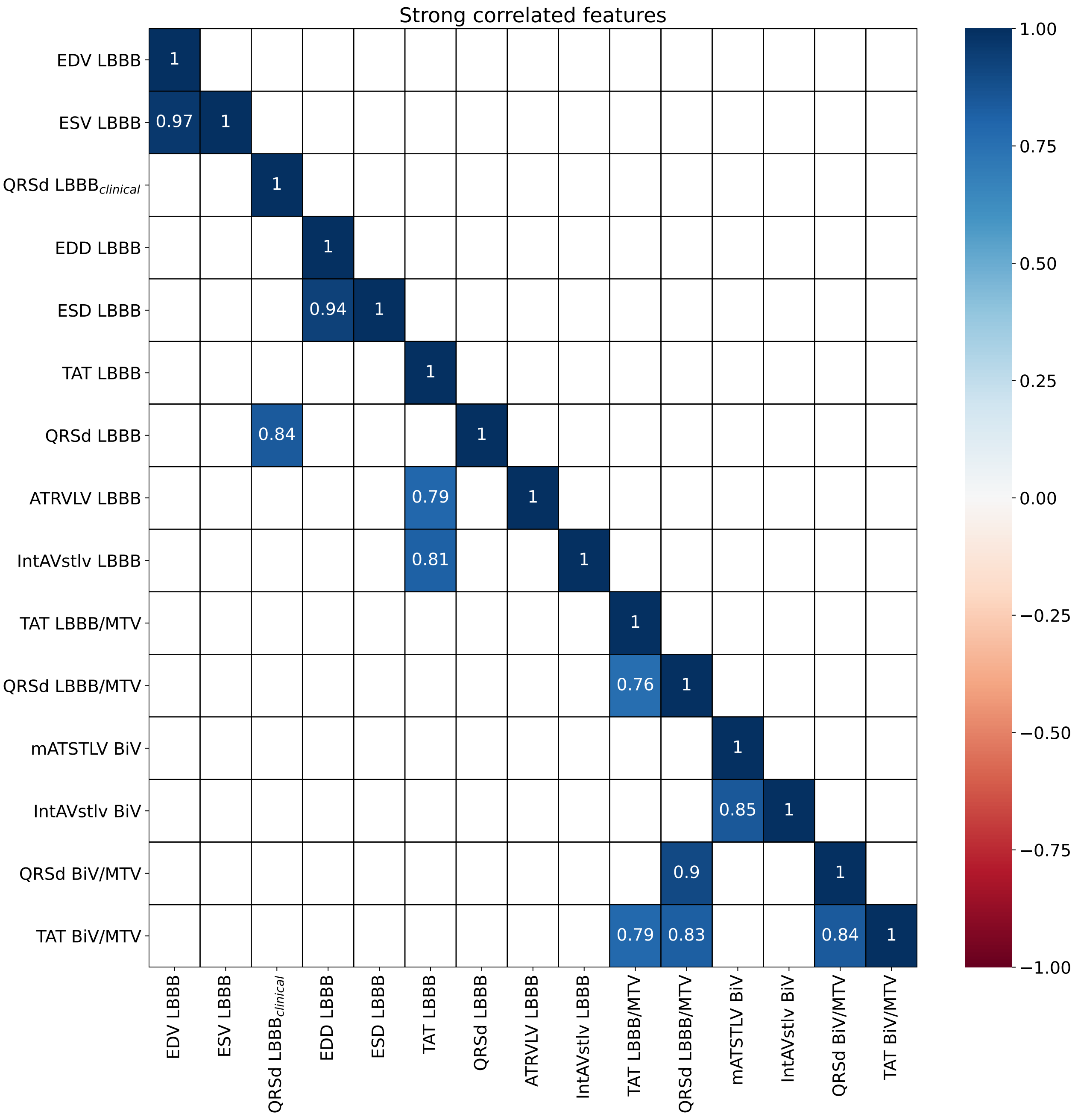

Figure S5. Correlation matrix of input features. Features with correlation coefficient r>0.75 and p<0.05 are shown.

List of abbreviations for figures:
LBBB indicates clinical indices before operation or model indices in LBBB mode, CRT - clinical indices after operation, BiV - model indices in BiV pacing mode.

Clinical indices

EDV - end diastolic volume;
EF - ejection fraction;
ESV - end systolic volume;
QRSd LBBB$_{clinical}$ - maximal duration of QRS complex on 12 leads;

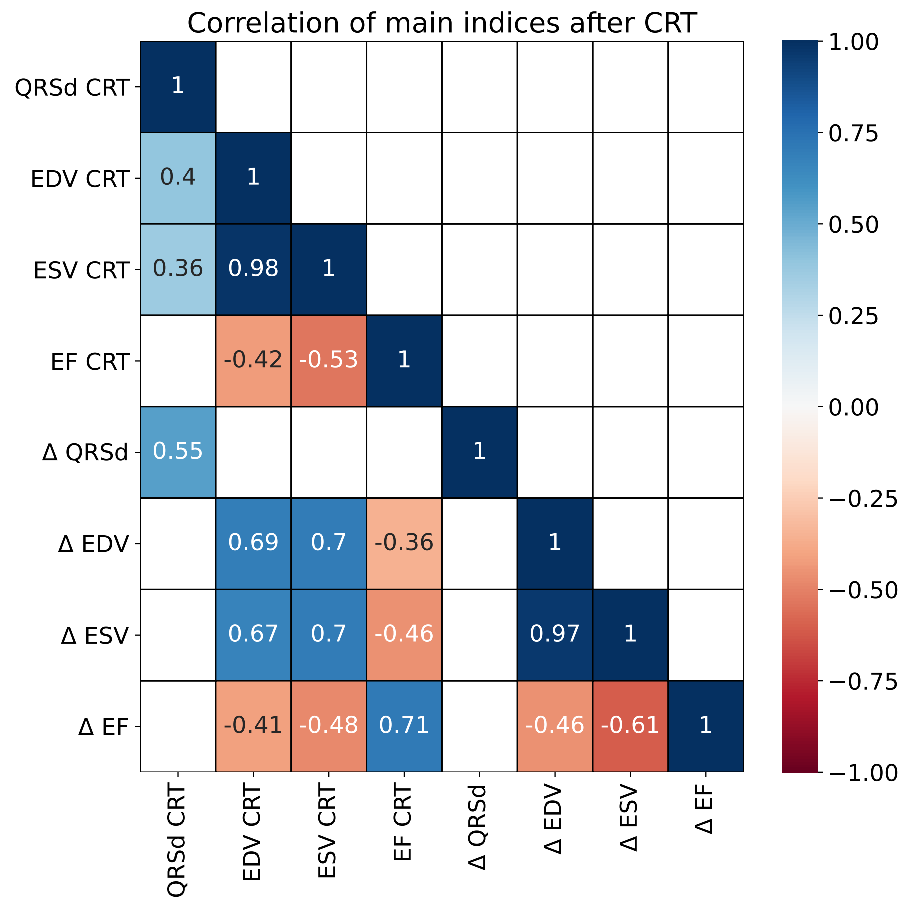

Figure S6. Correlation matrix of output features.

EDD - end diastolic diameter;
ESD - end systolic diameter;

Model indices

TAT - total ventricular activation time;
QRSd - maximal duration of QRS complex on 12 leads;
AT_{RVLV} - difference of total LV and RV activation time;
$IntAV_{STLV}$ - integral index of LV free wall and septum myocardial activation dyssynchrony;
mAT_{STLV} - difference between mean activation time of LV free wall and septum;
MTV - myocardial tissue volume;

CT/MRI indices

InfarctV - damage area (infarct/fibrosis) volume;
DLvRv - distance between active poles of LV and RV leads;
DLvLATZ - distance between LV lead and LAT zone;
DLvInfarct - distance between LV lead and infarct/fibrosis area;

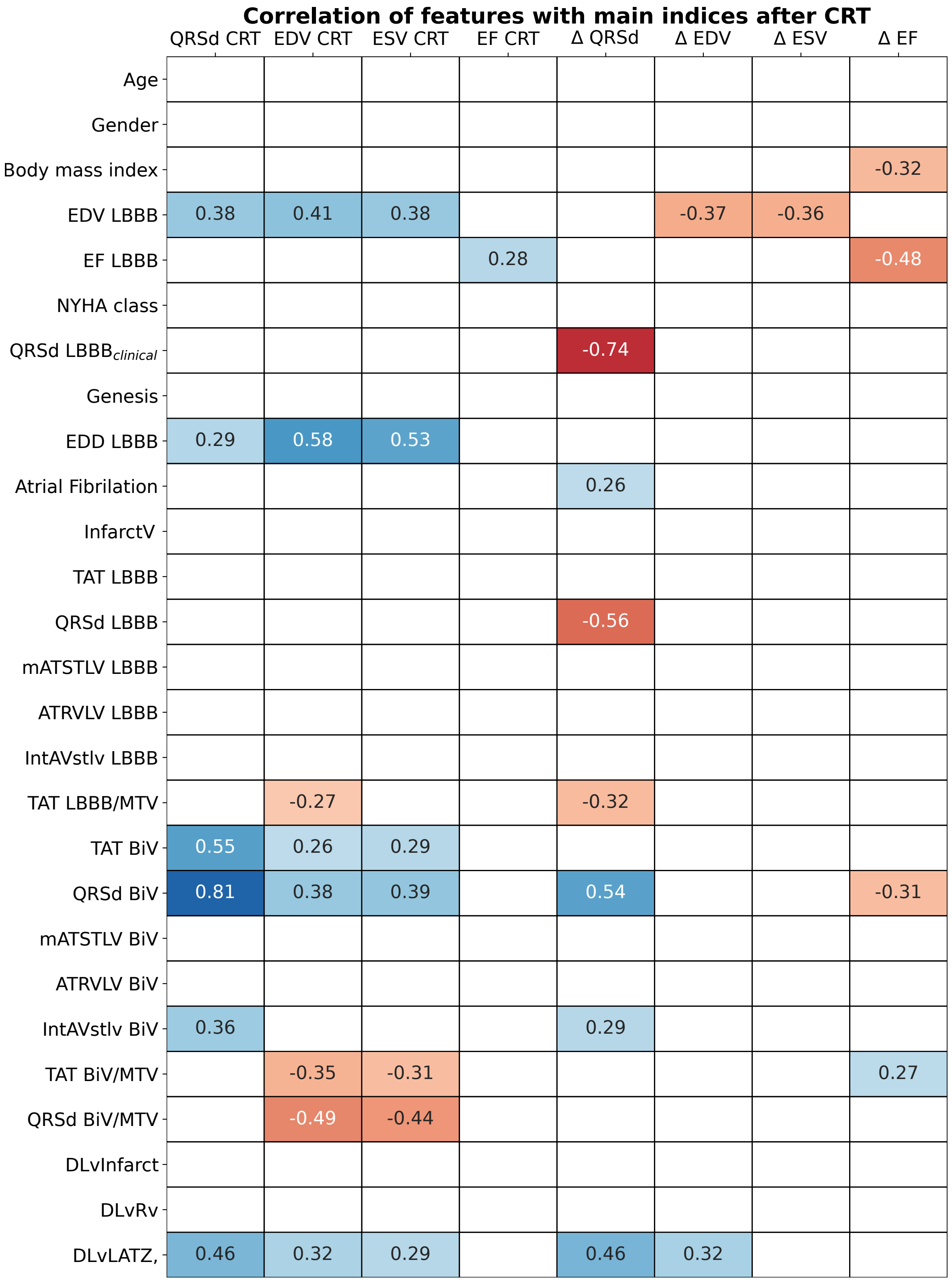

Figure S7. Correlation matrix between input and output features.

S.6 Logistic Regression Classifiers for ΔEF>10% definition of CRT response

Table S2. Coefficients of Logistic Regression on hybrid and clinical dataset for EF10 criterion of CRT response. Note all patients have LBBB aetiology and epicardial left ventricular lead.

Feature	Hybrid dataset	Feature	Clinical dataset
DLvInfarct,mm	1.00	EF LBBB, %	-1.09
EF LBBB, %	-0.98	NYHA stage	-0.41
TAT BiV/MTV	0.85	Gender	-0.21
DLvLATZ,mm	-0.77	EDD, mm	0.14
BMI	-0.69	Atrial Fibrillation	0.09
TAT LBBB/MTV	-0.40	QRSd LBBB, ms	-0.08
NYHA stage	-0.34	Genesis	-0.07
QRSd BiV, ms	-0.10		

S.7 Principal component analysis (PCA) in supervised and unsupervised stratification of CRT patients

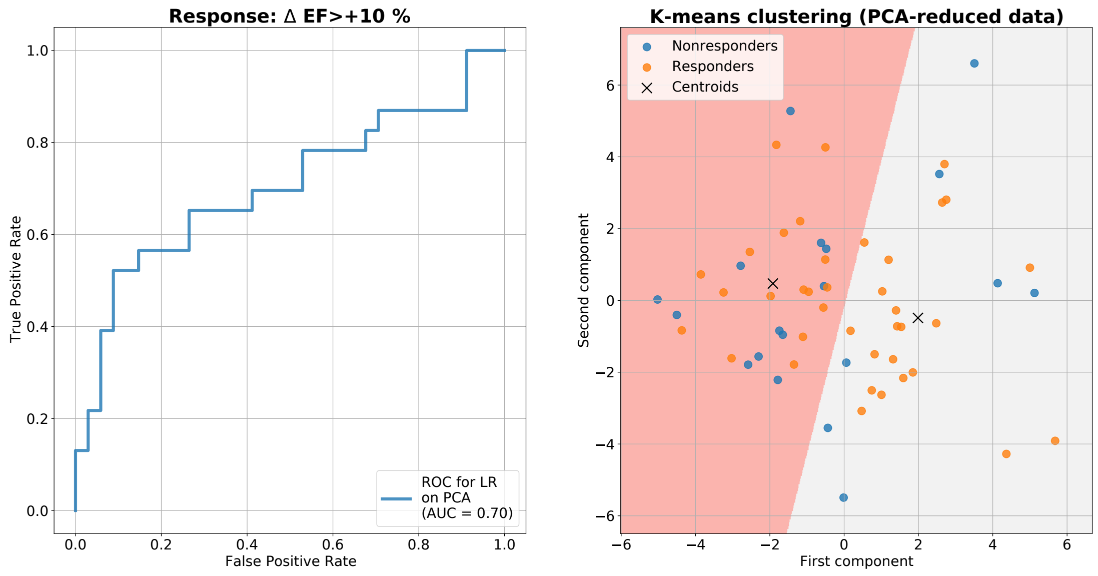

Figure S8. Results of using principal component analysis (PCA) in supervised and unsupervised stratification of CRT patients. Left: ROC curve for Logistic Regression model built on 5 PCA components. Right: k-means clustering on 2 PCA components (colors indicate two groups). Dots show respondres and responders defined by the EF10 criterion.

S.8 Classification based on ΔESV<-15% definition of CRT response

A summary of the statistics for the hybrid dataset labelled according to the ESV15 definition of responders and nonresponders is presented in Table S3. While testing various combinations of ML classifiers with feature selection approaches, we developed the best Linear Discriminant Analysis classifier with a Univariate feature selection algorithm showing at Leave-One-Out cross-validation the following: ROC AUC 0.74 (see Fig. S9), accuracy 0.70, sensitivity 0.87, specificity 0.37, positive prediction value(ppv) 0.73 and negative prediction value(npv) 0.58. We also compared the results of in Leave-One-Out cross-validation with more robust Five-Fold cross-validation, which provided similar qualitative results with the same best classifier showing comparable performance with ROC AUC 0.68±0.17, accuracy 0.67, sensitivity, 0.76, specificity, 0.47, ppv 0.75 and npv 0.51 (see Table S5). The results are slightly less powerful as compared with the best classifiers built on the EF10 criterion, showing higher ROC AUCs, similar sensitivity, but higher specificity as compared to ESV15 (Table S5). The ML scores based on ESV15 labelling are higher than compared with EF10 scores (0.69±0.18 *vs* 0.40±0.35, p<0.01, respectively) tending to overestimate predictions for the negative response (0.73±0.16 in responders *vs* 0.60±0.1 in nonresponders, p<0.001, for ESV15 against 0.65±0.33 and 0.24±0.26, p<0.001, for EF10; and p<0.001 between nonresponders for ESV15 *vs* EF10 criterion). Note that for both CRT response criteria, the average scores are significantly higher in responders *vs* nonresponders, indicating good predictive quality of the ML classifiers.

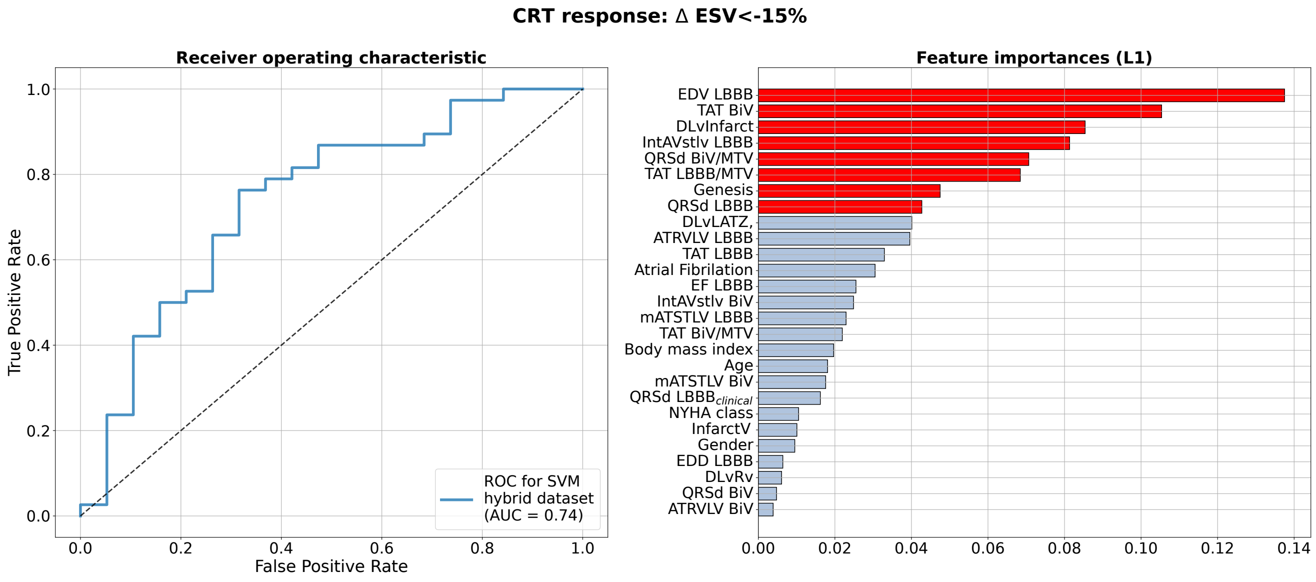

Figure S9. Best Machine Learning Classifier for CRT response prediction from the hybrid dataset of clinical and model-drived data for 57 patients. **Left panel** show receiver operating characteristic (ROC) curve for the best classifier (Support Vector Machine) based on the ΔESV<-15% criterion of CRT response (blue lines) using Leave-One-Out cross-validation. Values of the area under the ROC curve (ROC AUC) for the model are shown on the panel. **Right panel** show clinical and model-drived feature list in descending order of importance ranged using L1 feature selection approach (based on a weight of Logistic Regression) for the best classifier.

Table S3. Clinical, imaging, model data for responders and nonresponders by ESV15 criterion.

Variable	Patient cohort n=57<br>Responders n=38 (67%)			Nonresponders n=19 (33%)		
**Clinical data**						
Gender (male/female)	25/13			13/6		
Age, year	63±6			64±8		
BMI	28±5			30±4		
IHD/DCM	22 (58%)/16(42%)			14 (74%)/5(26%)		
History of AF	7 (18%)			5 (26%)		
	**LBBB**	**CRT**	**Δ, %**	**LBBB**	**CRT**	**Δ, %**
FC CHF :	decrease in FC 25 (66%)			decrease in FC 6 (32%)		
I	0 (0%)	7 (18%)**	7	0 (0%)	3 (16%)	3
II	16 (42%)	24 (63%)*	8	8 (42%)	7 (37%)	7
III	22 (58%)	2 (5%)**	-20	11 (58%)	4 (21%)*#	-18
QRSd, ms	192±23	143±17**	-25±12	189±26	146±22**	-20±17
**Echocardiography data**						
EDV, ml	307±87	199±69**	-33±21	270±101	309±161##	12±28##
ESV, ml	227±72	123±49**	-43±20	195±84	229±137##	14±32##
EDD, mm	73±7	64±9**	-13±11	73±8	70±10*#	-4±7##
ESD, mm	63±8	50±12**	-20±18	62±11	59±10*#	-5±9##
EF, %	26±6	38±6**	12±8	27±7	30±8##	2±6##
IVD, ms (n=34)	70±19	42±20**	-37±29	67±19	31±13*	-49±24
ΔTs, ms (n=34)	82±36	66±35*	-16±73	91±48	63±35	-15±49
SD12, ms (n=34)	31±14	24±12*	-16±71	34±17	24±13	-20±41
**CT MRI imaging data**						
MTV,ml	330±123			377±143		
InfarctV, ml	44±39			62±36#		
InfarctV/MTV	0.14±0.10			0.17±0.12		
DLvRv,mm	102±23			99±27		
DLvLATZ,mm	43±19			57±32		
DLvInfarct,mm	37±29			23±22		
**Model data**						
	**LBBB**	**BiV**	**Δ,%**	**LBBB**	**BiV**	**Δ,%**
TAT, ms	263±109	137±34**	-46±19	241±145	145±52**	-43±26
QRSd, ms	190±22	147±20**	-27±17	186±24	151±31**	-23±18
AT_{RVLV}, ms	101±65	23±23**	-73±27	93±89	21±24**	-81±18
$IntAV_{STLV}$, ms	100±51	34±15**	-55±32	112±70	33±17**	-48±66
mAT_{STLV}	0.35±0.09	0.28±0.13	-7±18	0.37±0.10	0.27±0.14*	-10±19
**Predictive model scores**						
Score by Feeny	0.63±0.20			0.55±0.23		
MLHD score(ESV15)	0.73±0.16			0.58±0.18##		

Mean±SD
* - p<0.05, ** - p<0.01 LBBB *vs* CRT or LBBB *vs* BiV. Comparisons between two dependent groups were made using Wilcoxon's test for quantitative data and McNimar's test for qualitative data.
# - p<0.05, ## - p<0.01 Responders *vs* Nonresponders. Comparison between two independent groups was carried out using the Mann-Whitney test for quantitative data and Pearson's chi-square test for qualitative data.
Δ - Average change in indicator ΔX = X CRT - X LBBB / XLBBB or ΔX = XBiV - X LBBB / X LBBB. Δ is calculated as the absolute difference for normalized values (EF and mAT_{STLV}) and FC

Table S4. Comparison of AUC for machine learning models by stratified five-fold cross-validation for different CRT response criterion

Classifier	Feature selection method					
	$\Delta EF > +5\%$			$\Delta EF > +15\%$		
	L1	MDA	Univariate	L1	MDA	Univariate
Logistic Regression	0.58 ± 0.17	0.65 ± 0.16	0.62 ± 0.16	0.63 ± 0.19	0.60 ± 0.19	**0.63 ± 0.19**
Linear Discriminant analysis	0.59 ± 0.18	0.66 ± 0.16	0.62 ± 0.16	0.63 ± 0.19	0.61 ± 0.19	0.62 ± 0.19
Support Vector Mashine	0.59 ± 0.18	0.63 ± 0.19	0.63 ± 0.17	0.62 ± 0.19	0.53 ± 0.20	0.61 ± 0.19
Random Forest	0.59 ± 0.17	**0.72 ± 0.15**	0.70 ± 0.16	0.55 ± 0.19	0.50 ± 0.20	0.55 ± 0.19
	$\Delta ESV < -15\%$			$\Delta EF > +10\%$ and $\Delta ESV < -15\%$		
	L1	MDA	Univariate	L1	MDA	Univariate
Logistic Regression	0.67 ± 0.17	0.61 ± 0.17	0.58 ± 0.18	0.69 ± 0.17	0.70 ± 0.15	0.73 ± 0.15
Linear Discriminant analysis	**0.68 ± 0.17**	0.62 ± 0.17	0.59 ± 0.18	0.67 ± 0.17	0.70 ± 0.15	**0.74 ± 0.15**
Support Vector Mashine	0.67 ± 0.17	0.58 ± 0.20	0.58 ± 0.18	0.68 ± 0.17	0.70 ± 0.16	0.73 ± 0.16
Random Forest	0.60 ± 0.17	0.56 ± 0.18	0.59 ± 0.18	0.66 ± 0.16	0.66 ± 0.16	0.69 ± 0.16

List of abbreviations for Tables S2, S3, S4:
BMI - Body mass index; IHD -Ischemic heart disease; DCM - Dilated cardiomyopathy; AF - Atrial Fibrilation; FC CHF- functional class of congestive heart failure; IVD - interventricular dyssynchrony; ΔTs - maximum temporary difference in peak systolic velocities between 12 LV segments; SD12 - standard deviation of the peak systolic velocities of 12 LV segments; MTV - myocardial tissue volume; DLvRv - distance between active poles of LV and RV leads; LAT - late activation time; DLvLATZ - distance between LV lead and LAT zone; DLvInfarct - distance between LV lead and infarct/fibrosis zone; TAT - total ventricular activation time; QRSd - maximal duration of QRS complex on 12 leads; AT_{RVLV} - difference of total LV and RV activation time; $IntAV_{STLV}$ - integral index of LV free wall and septum myocardial activation dyssynchrony; mAT_{STLV} - difference between mean activation time of LV free wall and septum; MLCD score (EF10) – ML score on the clinical data for EF10 criterion; MLHD score (EF10) - ML score on the hybrid data for EF10 criterion; MLHD score (ESV15) - ML score on the hybrid data for ESV15 criterion; L1 - Logistic Regression feature selection; MDA - Mean Decrease Accuracy; Univariate - Univariate statistical testing: two-sample t-test for continuous variables and chi-squared test for categorical variables;

Table S5. Average response prediction performance for best model in five-fold cross-validation

Responce definition	Accuracy	Sensitivity	Specificity	ppv	npv
$\Delta EF > +5\%$	0.71	0.87	0.41	0.74	0.62
$\Delta EF > +10\%$	0.74	0.65	0.79	0.70	0.78
$\Delta EF > +15\%$	0.69	0.33	0.80	0.32	0.80
$\Delta ESV < -15\%$	0.67	0.76	0.47	0.75	0.51
$\Delta EF > +10\%$ and $\Delta ESV < -15\%$	0.70	0.60	0.77	0.64	0.76

ppv - positive predictive value;
npv - negative predictive value;

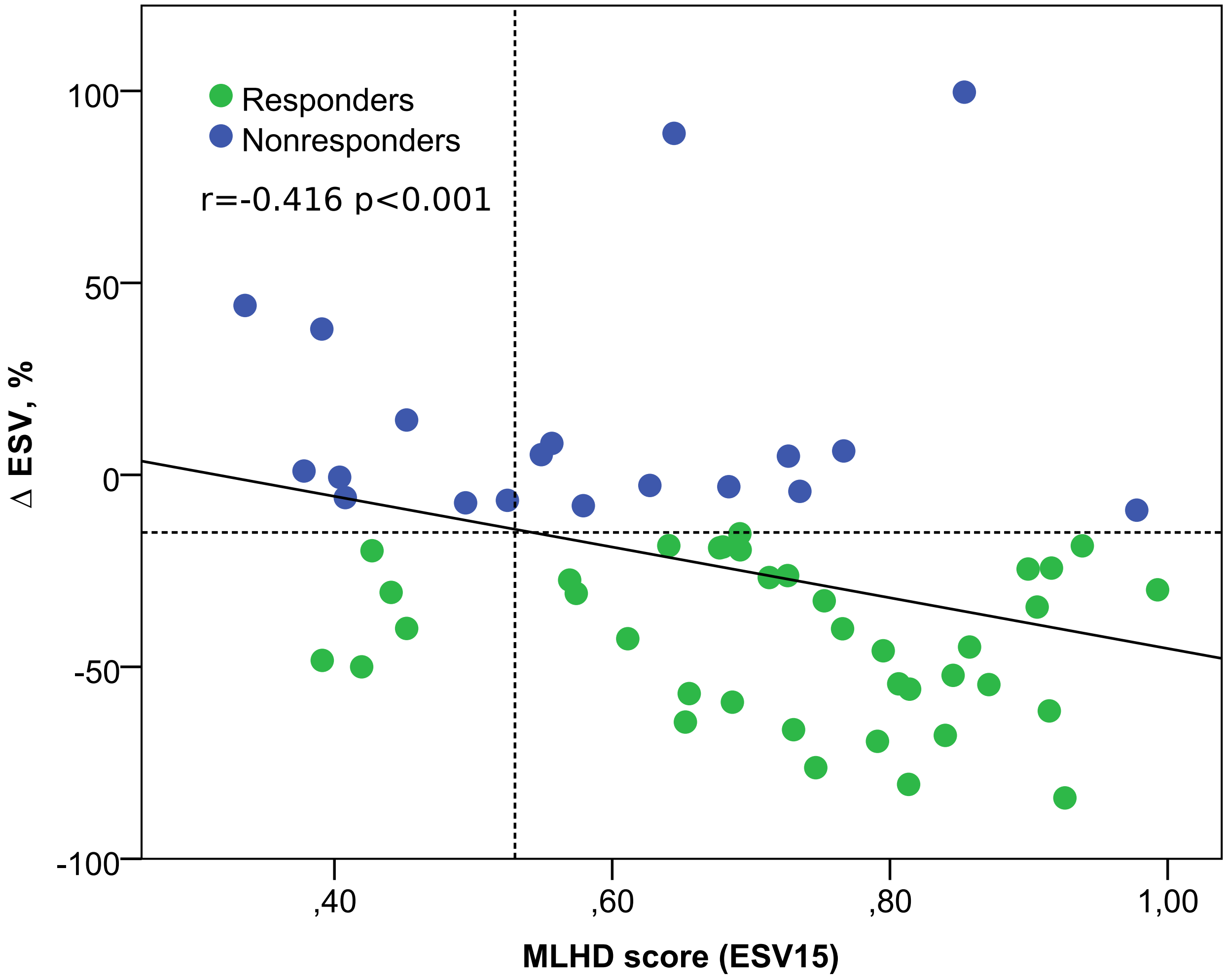

Figure S10. Relation between the ML score on the hybrid data for ESV15 criterion and the post-operational change in the ESV.Solid line - regression line Δ ESV = 21 − 66 MLHD score(ESV15); horizontal dotted line is threshold for ESV15 criterion equal to -15%; vertical dotted line is threshold for MLHD score (ESV15) equal to 0.53; r is the Spearman correlation coefficient; p is the significance of the difference between the correlation coefficient from zero.
